# Integrated On-Chip 3D Vascular Network Culture under Hypoxia

**DOI:** 10.3390/mi11050475

**Published:** 2020-04-30

**Authors:** Miguel Ángel Olmedo-Suárez, Tomohiro Sekiguchi, Atsushi Takano, Maria del Pilar Cañizares-Macías, Nobuyuki Futai

**Affiliations:** 1 Departamento de Química Analítica, Facultad de Química, Universidad Nacional Autónoma de México, Av. Universidad 3000, Ciudad de Mexico 04510, Mexico; miolsu22@hotmail.com (M.Á.O.-S.); pilarm@unam.mx (M.d.P.C.-M.); 2 Department of Mechanical Engineering, College of Engineering, Shibaura Institute of Technology, 3-7-5 Toyosu, Koto-ku, Tokyo 135-8548, Japan; md18048@shibaura-it.ac.jp; 3 Digital Manufacturing and Design Centre, Singapore University of Technology and Design, 8 Somapah Road, Singapore 487372, Singapore; cookdo812@hotmail.com

**Keywords:** 3D cell culture, hypoxia, angiogenesis, portable cell culture device, vascular network, normoxic/hypoxic transition

## Abstract

We developed a portable device made of poly(dimethylsiloxane) (PDMS)/polymethylmethacrylate (PMMA) for long-term 3D cell culture of vascular endothelial cells for the development of a vascular network and evaluated the device under different transitions between normoxia and hypoxia with good optical accessibility. The combination of a nested reservoir device and a bicarbonate/ascorbate buffer system accomplished on-chip incubation with 4.91 ± 0.86% pO_2_ and 5.19 ± 1.70% pCO_2_ for up to 10 days. Seventy-two hours of normoxic incubation preceding hypoxic culture increased the cell viability, network formation, and size and stability of the resulting lumens compared with those completely maintained in normoxia for the same total duration. We employed different parameters of the network (e.g., total mesh area, total length, number of branches, among others) for the comparison of different oxygen treatments in the device. The differential effect of hypoxic conditions based on the maturity of the vessels may be used as an external factor to improve vascular development in vitro.

## 1. Introduction

The vascular network formed by endothelial cells maintains tissue homeostasis by delivering oxygen and nutrients to cells and removing waste products. Thus, functional vasculature is essential to the clinical success of engineered tissue constructs, representing a challenge for regenerative medicine [1]. Experimental models of vascularization are used to provide new insights into vascular function, to elucidate details of molecular signaling pathways, and to serve as a platform for the development of new therapeutics against vascular targets [2]. In addition, the advent of organoids has generated recent interest in vascularization in vitro. In organoids, cells should be reorganized into complex tissue-specific structures. However, a major limitation in typical organoids is the lack of a structured vascular network that allows exchange of oxygen, nutrients, and waste. For example, upon reaching a certain size, organoids cease to proliferate and develop a necrotic core owing to limited diffusion of oxygen, nutrients, and metabolites. In this regard, the strategy of in vitro spontaneous vascularization in 3D constructs is of interest for efficient exchange of oxygen and nutrients in organoid cultures in order to develop models with in vivo-like functionality [3].

Hypoxia (oxygen level lower than 21%) is important to mimic the environment of cells under physiological conditions [4]. Particularly, hypoxia is of interest for a primary regulator for the development of a vascular network [5]. Endothelial cells and their lumenized networks are sensitive to hypoxia [6] involved in different diseases such cardiovascular [7], inflammatory [8], tumorigenesis [5,9], and microvascular damage associated with aging [10,11]. Hypoxia also modulates vessel patterning, maturation, and function, such as increased blood perfusion, owing to hypoxia-induced vasodilation [5,12], mainly by upregulating multiple pro-angiogenic pathways that mediate endothelial, stromal, and vascular support [5,13,14], as well as vascularization, during both embryonic development and adult life [5,15]. However, the evaluation of the hypoxia effect on self-assembled lumens in vitro is required because the vascular assembly and growth are differentially affected by hypoxic conditions based on the maturity of the vessels [16]. The effect of hypoxia on preformed vascular networks is important because there have been other recent approaches that evaluate the vascular promoting effect of hypoxia with structures previously assembled [17]. Novel research on the effect of hypoxia on both vasculogenesis and the resulting vasculatures, a portable and low-learning-cost system, and an optically accessible 3D culture under controlled oxygen concentrations are required.

Common and even microfluidic methods used to achieve hypoxia fall into three approaches [18]: (i) multi-gas incubators, gas tight chambers, or microchannels introducing N_2_/O_2_/CO_2_ gases [19,20]; (ii) addition of oxygen scavenging reactions (sodium sulfite [21], pyrogallol [21,22], water electrolysis [23], cellular metabolism [23,24,25]); and (iii) biochemically inducing a state of pseudo-hypoxia with compounds, such as CoCl_2_, that stabilize hypoxia inducible factors (HIFs) under normoxic conditions [26,27]. However, these methods have the following problems: (i) they require bulky gas supplies and tubing, and thus compromise easy handling and optical access; (ii) the reactions alter the medium compositions and further affect cellular responses [25,28]; and (iii) CoCl_2_ influences the transcription of the distinct sets of genes that were not affected by real hypoxia [26,29]. Therefore, a simple hypoxic culture system capable of maintaining the long-term physicochemical properties of 3D culture is still challenging.

We developed a standard, cover glass-sized device made of poly(dimethylsiloxane) (PDMS)/ polymethylmethacrylate (PMMA) for long-term 3D vascular network formation under controlled O_2_ and CO_2_ levels using a bicarbonate/sodium ascorbate (NaHAsc) buffer system. We then demonstrated the capability of this system to culture endothelial cells in hydrogels and develop vascular networks. The control of oxygen concentration in the culture was adequate to evaluate the organization of vascular networks under different transitions of normoxia/hypoxia and the relationship between the duration of normoxic/hypoxic incubation stages and angiogenic parameters (tube length, number of junctions, meshes, and isolated elements). We also evaluated the effect of preformed vascular networks under hypoxia exposure for short and prolonged durations. Macroscale culture systems as other microfluidic systems require external sources to control the conditions of culture. Our device is one of the simplest standalone systems for 3D cell culture under adjustable hypoxia conditions as it incorporates a source of CO_2_ and an O_2_ scavenger within the device. This offers the following advantages: (1) maintaining portable cell culture as a closed system requiring only a heat source as the external activator; (2) cells or lumens can be observed under a non-specialized microscopic stage without disturbing the atmosphere around the culture; (3) high incubation capacity compared with gas exchange between microchannels; (4) no need for conditioning gases, gas chambers, or other bulky and expensive equipment; and (5) no obstacle in the light path, unlike an external incubator or chamber.

In addition, this device provides an easy protocol of seeding and maintenance of cells’ 3D vascular network formation; we introduced a fibroblast-conditioned medium as a simple alternative to a co-culture-based angiogenesis induction method.

## 2. Materials and Methods

### 2.1. Device Construction

**Figure 1:**
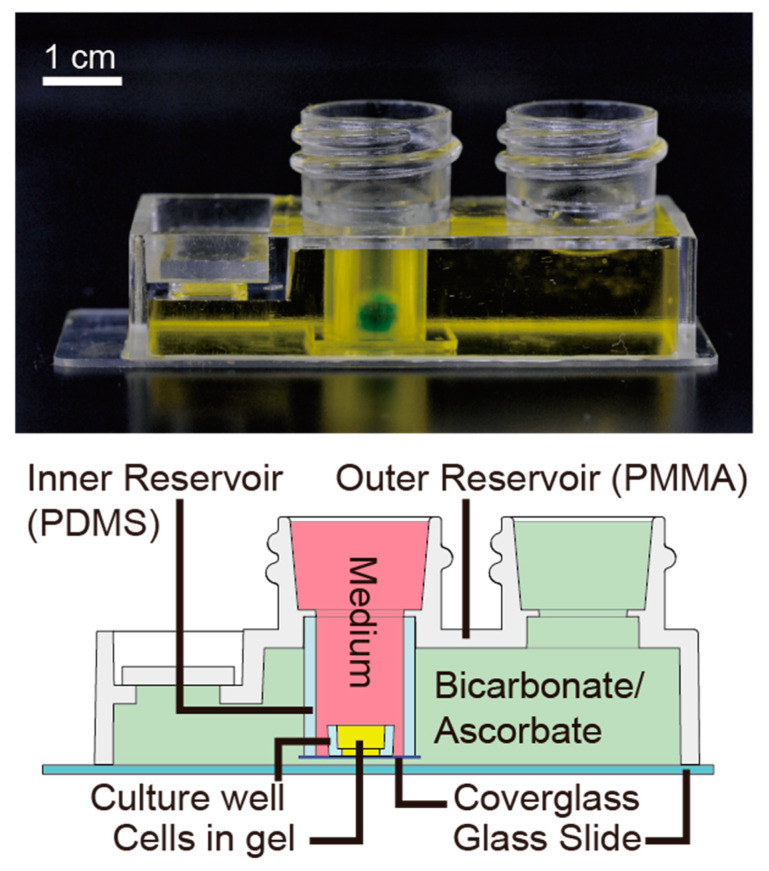
Portable device for the development or vascular networks under controlled oxygen and carbon dioxide concentrations. The device consists of two reservoirs: the inner reservoir where cells are cultured inside a fibrin-collagen hydrogel at the bottom, and the outer reservoir made of polymethylmethacrylate (PMMA) for the bicarbonate buffer/ascorbate solution. These reservoirs are divided by a tubular wall made of poly(dimethylsiloxane) (PDMS), where gas (O_2_ and CO_2_) and moisture exchange occurs.

Figure 1 shows a device for hypoxic 3D culture without any external gas supply. The device has a pair of nested reservoirs: (1) an inner reservoir for cell culture and culture media and (2) an outer reservoir for a bicarbonate and ascorbate buffer solution. The sidewall of the inner reservoir made of a cylindrical PDMS tube allows for gas exchange by diffusion. The enclosure is made of PMMA and has two threaded necks for each of the two reservoirs. The outer reservoir accommodates a jacket solution to allow gases to diffuse into the inner reservoir. The inner reservoir has one circular culture well at the bottom.

The fabrication processes of the cell culture device are summarized in Figure S1. A PDMS well for 3D gel culture (ID, H = 4, 3.3 mm) was fabricated by casting KE106 (Shin-Etsu Chemical, Tokyo, Japan) from a 3D printed mold. The mixing ratio of the base and curing agent when making the PDMS of the well was 10:1. The PDMS was cured by placing at 65 °C overnight. The PDMS well and a PDMS tube (ID, OD, H = 10, 12, 15 mm, Kyowa Kogyo, Saitama, Japan) were bonded to a cover glass (18 × 18 mm No. 1, Matsunami, Osaka, Japan) after plasma treatment (20 mA, 20 Pa, 30 s, room temperature) (SC-708, Sanyu Electron, Tokyo, Japan). The PDMS–coverglass assembly was attached to an injection molded reservoir made of PMMA (Proto Labs G.K., Kanagawa, Japan) with silicone adhesive to form a nested reservoir pair. The reservoir pair was bonded onto a glass slide (S9111, Matsunami, Osaka, Japan) with silicone adhesive. We can provide actual molded parts upon request (http://www.cd.mech.shibaura-it.ac.jp/).

The entire device was stored at room temperature for at least 24 h prior to use. Screw caps (5183-4303, Agilent, Santa Clara, CA, USA) were used to prevent oxygen or any external contamination from the atmosphere. Caps used for the inner reservoir were perforated and a round cover glass (φ15 mm, No. 1, Matsunami, Osaka, Japan) was bonded with silicone adhesive to allow optical access to the inside of the PDMS well.

### 2.2. Device Characterization

The O_2_ and CO_2_ concentrations inside the inner reservoir were measured using a mock device that does not contain a PDMS well. To measure the partial O_2_ pressure (pO_2_), the fluorescence quenching-based oxygen probe with temperature compensation (FOSPOR-T1000-TS-NEO, Ocean Insight, Largo, FL, USA) coupled to a fluorimeter (NeoFox-GT, Ocean Insight, Largo, FL, USA) was used. The fluorimeter was calibrated with air saturated deionized water and a 1 M sodium sulfite solution for 0% and 20.9% pO_2_. The outer reservoir was filled with 14 mL of 0.001–1 M sodium L-ascorbate (NaHAsc) (58049-17, Kanto Kagaku, Tokyo, Japan), 0.8 M NaHCO_3_ (37116-00, Kanto), and 65 mM Na_2_CO_3_ (196-01595, Wako, Osaka, Japan) in deionized water; the inner reservoir was filled with 1.5 mL of 10 mM NaHCO_3_. The oxygen probe was placed at the inside bottom of the inner reservoir through a perforated cap. The device was maintained at 37 °C using a hotplate and the pO_2_ in the inner reservoir was recorded every minute for up to 90 h.

The partial CO_2_ pressure (pCO_2_) of the inner reservoir was obtained from the measured pH of 1.5 mL of 10 mM NaHCO_3_ in the inner reservoir. The outer reservoir was filled with 14 mL of NaHCO_3_, Na_2_CO_3_, and 1.0 M NaHAsc solution in deionized water. The pH and temperature of the inner reservoir were measured with a pH meter (LAQUA act, Horiba, Tokyo, Japan). The pCO_2_ was calculated considering the chemical equilibria previously described in a CO_2_–HCO_3_^−^–CO_3_^2−^ system [30].

### 2.3. Cell Culture and Vascular Network Development under Normoxic–Hypoxic Transition

Human umbilical vein endothelial cells (HUVECs) (CC-2519, Lonza, Basel-Stadt, Switzerland) and human lung fibroblasts (hLFs) (CC-2512, Lonza) were maintained in endothelial cell growth medium-2 (EGM-2, Lonza). For HUVECs and hLFs, passages 4–6 were used. When HUVECs reached 80–90% confluence, they were detached with Accutase (AT-104, Innovative Cell Technologies, Inc., CA, USA), pelleted by centrifugation at 100 *g*, and resuspended in Hanks’ balanced salt solution (HBSS) (084-08345, Wako). Cells were counted using an automatic cell counter (Scepter 2.0, Millipore, Burlington, MA, USA) to adjust the cell seeding density to (0.5–2) × 10^5^ cells/well.

HUVECs were suspended in 40 µL of HBSS without phenol red (085-09355, Wako). Then, 10 µL of 25 mg/mL fibrinogen (F8630, Sigma, St. Louis, MO, USA) and 2 µL of 3 mg/mL collagen type I (ASC-1-100-20, Nippi, Tokyo, Japan) were added to the suspension. The PDMS well was filled with the cell suspension immediately after the addition of 0.5 µL of 50 U/mL thrombin (T4648, Sigma) and incubated for 10 min at room temperature to allow for complete gelation. To achieve vascular network formation, 1.5 mL of hLF-conditioned medium (EGM-2 exposed in hLF cells for at least 24 h) was added. The outer reservoir was filled with bicarbonate buffer solution (0.8 M NaHCO_3_ and 65 mM Na_2_CO_3_) and the reservoir was capped tightly.

For normoxic experiments, the devices were incubated in a buffer solution without NaHAsc, cultured for 240 h (10 days), changing the buffer solution every 72 h, and evaluated daily using a microscope. For hypoxic experiments, the devices were pre-incubated for 24 h, 36 h, and 72 h at 37 °C in bicarbonate buffer without NaHAsc (normoxic preincubation), and then switched to a hypoxic condition by switching to bicarbonate buffer containing 1 M NaHAsc. The buffer solution was changed every 72 h and maintained for seven days. Cells cultured in normoxia and subsequent hypoxia were compared with cells cultured only with normoxia for the same total cultivation time. Brightfield images were captured using an inverted microscope (EVOS XL Core Cell Imaging System, Thermo Fisher Scientific, Waltham, MA, USA). Quantitative analysis of the vascular networks was realized with an Angiogenesis Analyzer [31]. The Angiogenesis Analyzer detects vascular networks and analyzes the vascular organization. The angiogenic parameters of cells cultured in normoxic–hypoxic conditions were compared with values obtained from cells cultured in only normoxia during the same period of culture (control group). The results are expressed as mean percent change relative to the control group. The parameters described were as follows: (1)number (#) of meshes: areas enclosed by segments. (2) Total mesh area: sum of areas enclosed by the segments. (3) # of segments: elements, which are delimited by two junctions. (4) Isolated elements: binary lines that are not branched. (5) Total length: sum of length of segments, isolated elements, and branches in the analyzed area. (6) Total segments length: sum of lengths of the segments in the analyzed area. (7) # of Branches: elements delimited by a junction and one extremity. (8) Total branching length: sum of length of the branches in the analyzed area. (9) Junctions: correspond to nodes or group of fusing nodes.

After hypoxic exposure for seven days, the cell viability was measured using a LIVE/DEAD cell staining kit II (PK-CA707-30002, PromoKine, Heidelberg, Germany). A working solution of 8 µM calcein-AM and 16 µM ethidium-1 homodimer (EthD-1) was prepared in HBSS without phenol red. The medium was removed from the inner reservoir, and the inside of the inner reservoir was washed twice with 1.5 mL of HBSS. Then, 150 µL of the working solution was added on the PDMS well. The devices were then incubated while being shielded from light at 37 °C for 30 min. Subsequently, the gel was removed from the well and placed in a glass slide. Fluorescence images were recorded with a fluorescence microscope (DMi8, Leica, Hesse, Germany). Each of the experiments was performed in triplicate.

## 3. Results

### 3.1. Characterization of O_2_ and CO_2_ Levels

**Figure 2:**
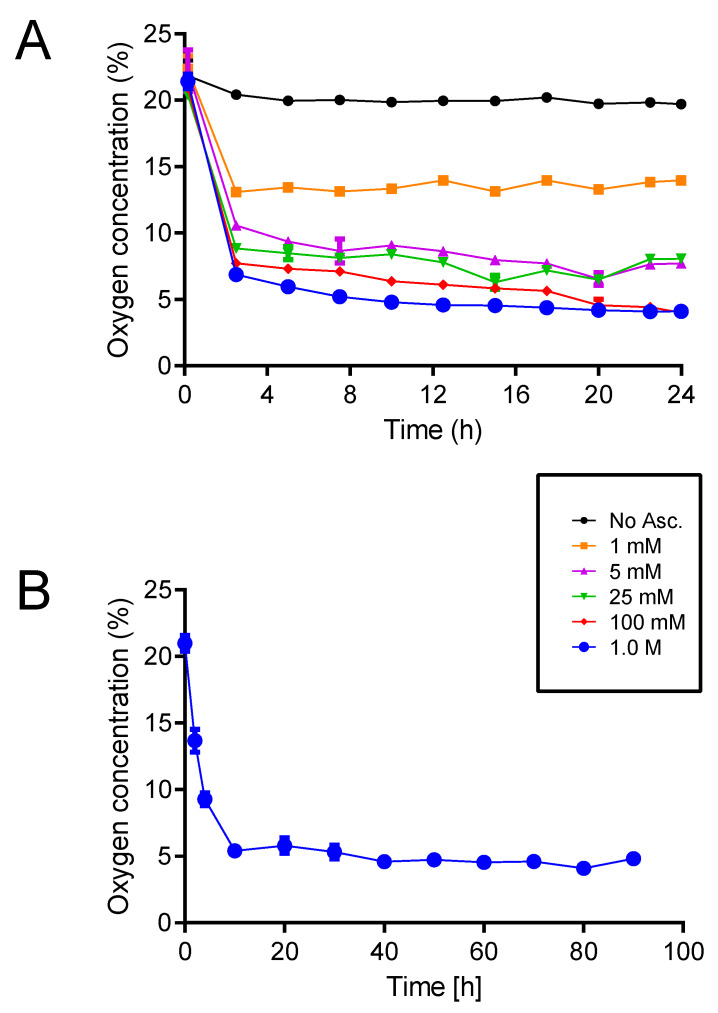
Oxygen concentrations measured within a device incubated with ascorbate buffer solutions of different concentrations (*N* = 3; mean 4.91 ± 0.86 standard deviation)). (**A**) The evaluation of a sodium ascorbate (NaHAsc) solution with different concentrations (0.001–1 M) for 24 h. The oxygen concentrations decreased proportionally with increasing NaHAsc concentration. (**B**) The time course of the oxygen levels of 1 M NaHAsc. The lowest level of oxygen in the device could be maintained up to 90 h.

Figure 2 shows the stability of O_2_ levels inside the inner reservoir when the bottom of the device was incubated at 37 °C. A certain decrease in the O_2_ levels by varying the concentration of ascorbate in the buffer solution was observed. The O_2_ levels could be adjusted in a range of 4.91%–20.9% by increasing the NaHAsc concentration in the range of 1 mM to 1 M (1000 times). Figure 2A shows that the concentration as high as 1 M of NaHAsc was needed to reach within 4.91% ± 0.86% pO_2_ within 2 h of incubation. When 1 M NaHAsc was used, as shown in Figure 2B, the O_2_ level was maintained in the range of 4.09% to 5.70% for up to 90 h.

Table 1 shows the pCO_2_ levels calculated from the pH measurements in the inner reservoir when the outer reservoir was filled with bicarbonate buffer with NaHAsc (hypoxic conditions) or without NaHAsc (normoxic conditions). The 0.8 M NaHCO_3_ and 65 mM Na_2_CO_3_ buffer solution in the outer reservoir was adequate to provide 5.19% ± 1.70% pCO_2_. The addition of sodium ascorbate to the buffer solution did not affect the pCO_2_. Therefore, the 0.8 M NaHCO_3_, 65 mM Na_2_CO_3_, and 1 M NaHAsc buffer solution was adequate for the on-chip culture with 5% pO_2_.

### 3.2. Network Formation in the Normoxic Condition

**Figure 3:**
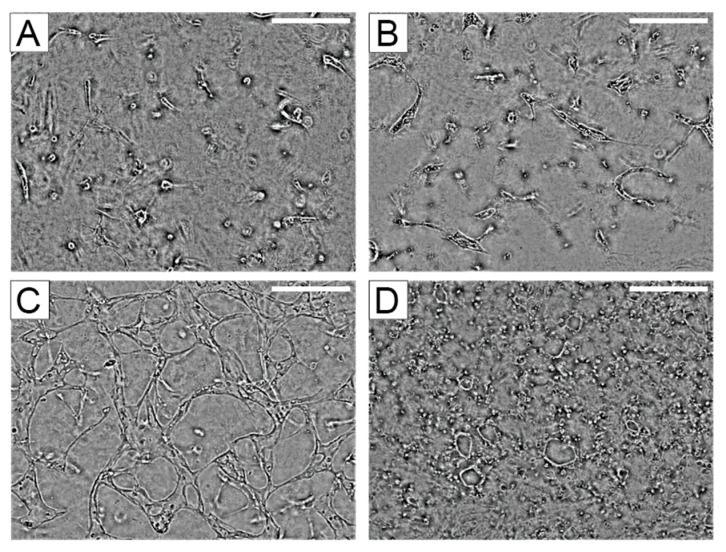
The vascular network formation depends on the number of cells seeded on a cell culture well in the device: (**A**) 0.5 × 10^5^ cells/well; (**B**) 1.0 × 10^5^ cells/well; (**C**) 2.0 × 10^5^ cells/well; (**D**) 2.5 × 10^5^ cells/well. All images were taken at 72 h of culture. Scale bar = 150 µm.

We cultured HUVECs seeded into fibrin/collagen gel at different densities and submerged them in hLF-conditioned EGM-2 medium to induce network formation. Figure 3 shows images of cells seeded with (0.5–2.5) × 10^5^ cells/well after culture for 72 h in normoxic conditions. As shown in Figure 3C, HUVECs seeded at a density of 2 × 10^5^ cells/well formed visible vascular networks. Alternatively, Figure 3A,B show that cell densities less than 2 × 10^5^ cells/well did not allow cell spreading or vessel forming. Figure 3D shows that HUVECs seeded at 2.5 × 10^5^ cells/well formed a large lumen that covered the whole view field. Therefore, we seeded HUVECs at (2.0 ± 0.1) × 10^5^ cells/well ((1.59 ± 0.07) × 10^4^ cells/mm^2^) for all experiments thereafter.

**Figure 4:**
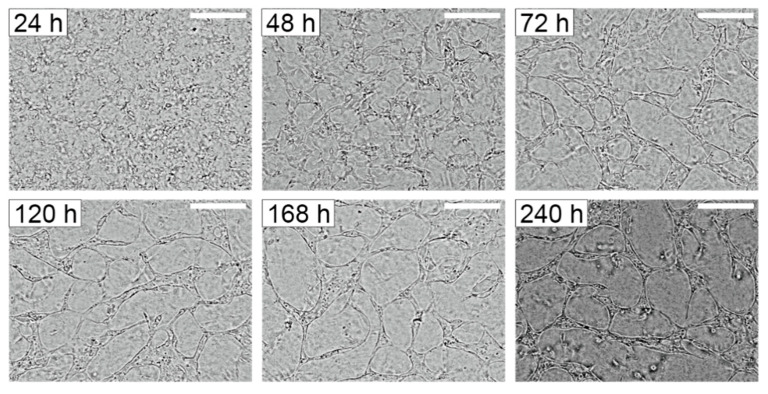
Development of vascular networks of human umbilical vein endothelial cells (HUVECs) formed within the device under normoxia for 240 h. Scale bar = 150 µm.

The on-chip network formation of HUVECs during normoxic incubation for ten days is presented in Figure 4. Sprouting of HUVECs was observed at Day 2. These sprouts were well lumenized at Day 3. Vascular networks were formed on-chip in 3D culture under normoxic conditions and could be maintained for at least 240 h.

### 3.3. Network Formation and Development in the Normoxic–Hypoxic Transition

**Figure 5:**
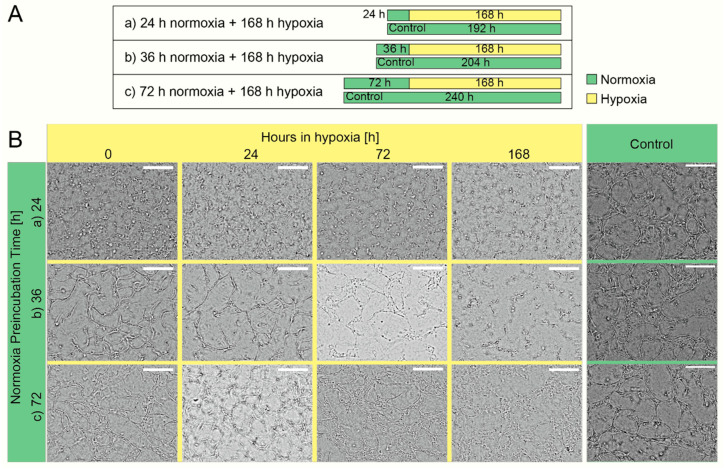
Vascularization under different conditions of preincubation in normoxia. (**A**) Time scheme of the cell culture at different preincubations in normoxia followed by hypoxia transitions. The cells are kept in preincubation in normoxia for 24, 36, and 72 h prior to the transition to hypoxic conditions for 168 h. Each condition was compared with cells maintained in normoxic conditions (Control). (**B**) Temporal evolution of the lumens at different normoxia preincubations. The lumenization increased with the time in normoxic preincubation. Scale bar = 150 µm.

We characterized the vascular network formation of HUVECs on-chip under hypoxia. First, we found that cells did not form any vessel, nor did they proliferate (data not shown) when the cells were subject to hypoxia from the beginning. As a result, we proposed the hypoxic culture with normoxic “preincubation”. As illustrated in Figure 5A, we first preincubated cells in normoxia (20.9% O_2_) for either 24, 36, or 72 h, and then the inside of the device was switched to hypoxia. Figure 5B shows that the preincubation in normoxia for 72 h allowed for better vascular network development compared with the normoxic preincubation for 24 or 36 h.

**Figure 6:**
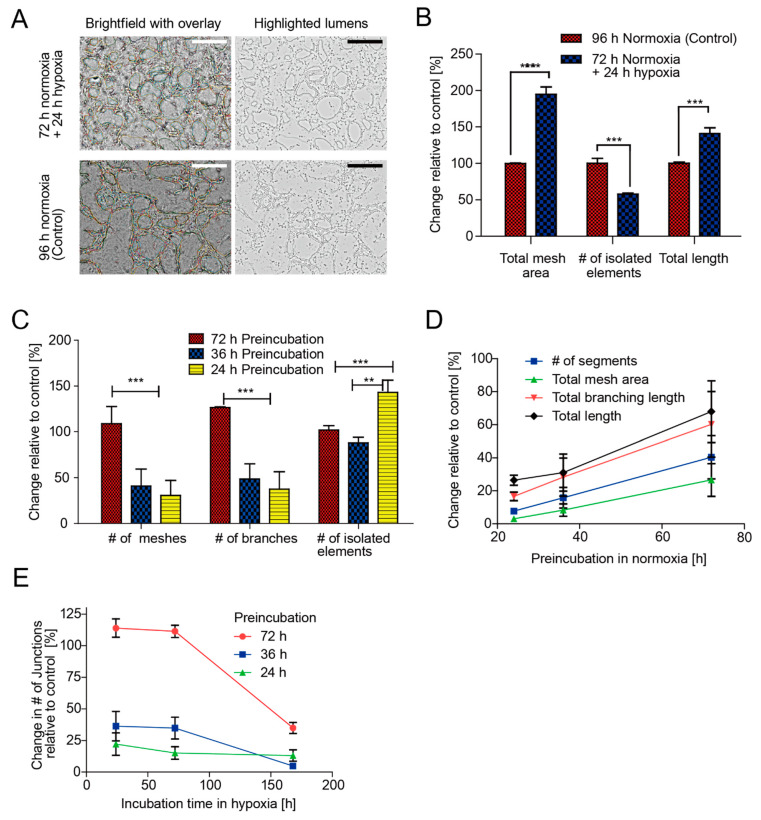
Angiogenic parameters of the vascular networks of HUVECs under different normoxic preincubations and followed by hypoxic transitions. Parameters increased with the time of normoxic preincubation and short periods of hypoxia exposure (24 h). Prolonged hypoxia decreased the angiogenic parameter values: (**A**) representative images of vascular networks of HUVECs cultured for 72 h normoxia/24 h hypoxia and control cells (96 h normoxia) (scale bar = 150 µm); (**B**) angiogenic parameters of cells cultured for 72 h normoxia/24 h hypoxia and control cells (96 h normoxia); (**C**) angiogenic parameters in cells preincubated in normoxic conditions for 24, 36, and 72 h and switched to hypoxia for 24 h; (**D**) relationship between preincubation time in normoxia and vascular network parameters; (**E**) effect of incubation in hypoxia on the relative change in the number of junctions. The standard deviation of the mean (standard deviation) is presented in all error bars, *N* = 3.

The effect of hypoxia and preincubation on the development and stability of vascular networks was evaluated using angiogenic parameter analysis, which allows a quantitative evaluation of the vessels-like network organization by extracting characteristic information of the network [31]. In Figure 6, lumens formed and incubated at 24, 36, and 72 h normoxia, followed by hypoxia up to 168 h, were compared with those incubated in normoxia for the same duration of culture (control; as shown in Figure 4). Changes in the angiogenic parameters shown in Figure 6B–E were expressed as the percentage relative to the control.

Figure 6A shows representative images of lumens formed after 72 h preincubation followed by hypoxia for 24 h, and those formed after normoxia for 96 h (control). Hypoxia for 24 h increased the cell density and branch density in comparison with the whole normoxic condition. Figure 6B shows the total mesh area (sum of the areas enclosed by segments), the number of isolated elements (binary lines not branched), and the total length of the vessel (sum of length of segments, isolated elements, and branches in the analyzed area) detected from pictures of lumens in the two cases referred to above. The total mesh area in the 24 h hypoxia case increased by 95%; the number of isolated elements decreased by 42% and the total length increased by 41% in comparison with the control. These results are in agreement with Nyberg and Grayson [17]; they determined, in a traditional system of culture, that hypoxia inhibits the vascular assembly of individual ECs, but promotes angiogenesis on preassembled lumens in an normoxic environment.

Figure 6C shows the number of meshes; branches (elements delimited by a junction and one extremity); and isolated elements for 24, 36, and 72 h of preincubation in normoxia followed by 24 h of hypoxia. These angiogenic parameters for lumens preincubated for 24 and 36 h and switched to hypoxia for 24 h were less than those of their respective controls with the exception of the number of isolated elements in which 24 h normoxia followed by 24 h hypoxia increased the number of isolated elements. These results suggest that the long preincubation before hypoxia promotes lumenization. Additionally, the lumens formed at 24 and 36 h of preincubation were less mature in comparison with the condition of 72 h, owing to a reduction in the number of meshes and branches. As shown in Figure 6D, the number of segments, total mesh area, total length, and total branching length (of the length of branches in the analyzed area) were proportional with the time of preincubation in normoxia. Figure 6E shows the relative changes in the number of junctions (nodes or group of fusing nodes) for various times of preincubation followed by hypoxia for different durations. The vessel development stopped for 24 and 36 h of preincubation. In addition, regardless of the preincubation time, a long exposure hypoxia (more than 72 h) destabilized the preformed vessel, as observed by the reduction in the number of junctions.

**Figure 7:**
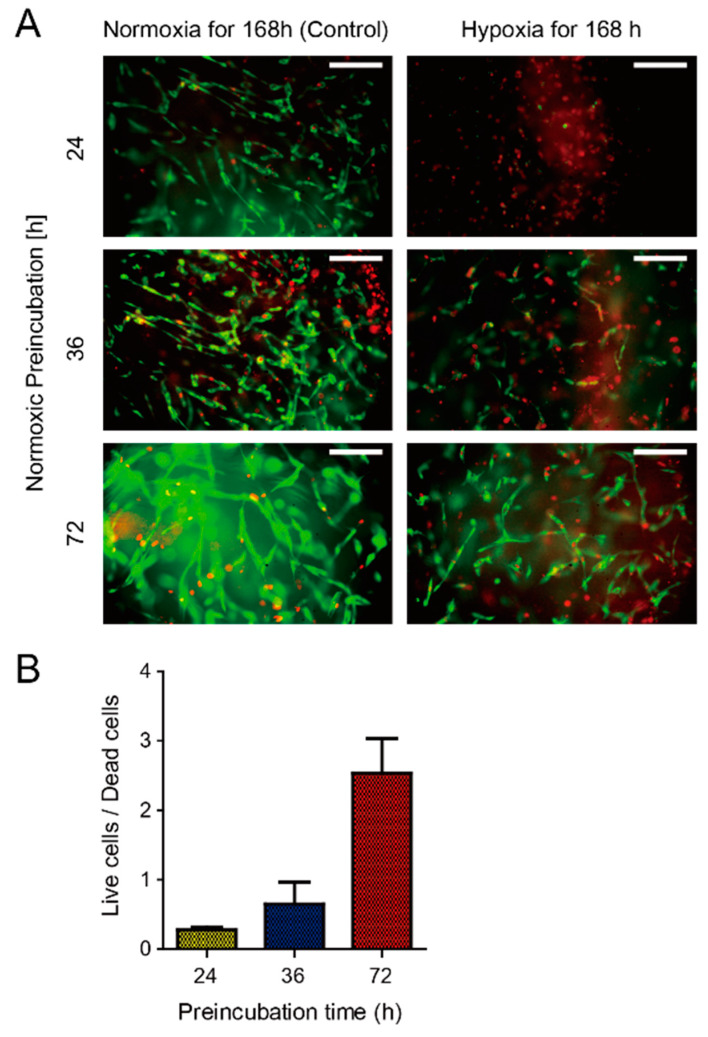
The effect of hypoxia and preincubation duration on the viability of HUVECs cultured on-chip: (**A**) fluorescence images of LIVE/DEAD stained HUVECs. For each image, either normoxia or hypoxia for 168 h followed normoxic preincubation for either 24, 36, or 72 h. Green represents live cells and red represents dead cells. Scale bar = 150 µm. (**B**) Ratio of live/dead cells as a function of preincubation time in normoxic conditions. This ratio is proportional to the time of normoxic preincubation.

Figure 7 shows the viability of HUVECs cultured on-chip for each period of normoxic preincubation followed by prolonged incubation. After keeping the cells either in normoxia or hypoxia for 168 h (seven days), the cell viability was measured with the LIVE/DEAD method. Figure 7A shows the fluorescence images of the HUVECs stained with the LIVE/DEAD reagents. For each condition of preincubation, the population of living cells incubated in normoxia was greater than cells cultured in hypoxia for 168 h. Moreover, as shown in Figure 7B, the ratio of live/dead cells was proportional to the time of preincubation in normoxia. The method LIVE/DEAD was successfully applied to evaluate the viability of the cells cultured in the devices. However, during the extraction procedure of the gel from the devices, the lumen structure was lost. Therefore, we could not observe the lumen structure by fluorescence.

## 4. Discussion

Our experiments demonstrate that it is possible to develop 3D vascular networks on a chip under normoxic/hypoxic atmospheres. From these findings, various questions can be raised about the fundamental molecular process or genes involved in this phenomenon. Of course, these issues are beyond the scope of this article, but propose a wide field to explore in the future.

Our 3D gel culture device with a nested reservoir pair is a simple option for modeling angiogenesis under hypoxia that has a low cost and allows for easy imaging. Storage of a CO_2_ source and a O_2_ scavenger inside of the device offers the following: (1) calibration-free CO_2_ incubation with or without hypoxia; (2) direct optical access even during hypoxic CO_2_ incubation, unlike systems requiring an external chamber; and (3) a simple, well-based 3D gel culture that is much easier to adopt than microfluidic 3D coculture methods such as capillary barrier-based segmentations of the cell culture area [32,33]. The simple design of this chip helped us provide a simple setup for a 3D vascular network formation assay with hypoxia without external apparatus for gas control or require expertise in microfluidics. The closed microsystem avoids gas exchange with the surrounding atmosphere; therefore, the gas composition of the incubator did not influence the performance of the device. We only verified that the incubator provides the optimal temperature for cell culture. Our miniature hypoxic culture device can incubate 3D cultures with variable oxygen levels down to 4.91% for up to ten days. The O_2_ and CO_2_ levels achieved within the device were similar to a previously developed on-chip incubation device [30]. However, our device offers an extended hypoxic incubation time, more repeatable and stable O_2_ and CO_2_ levels, and reduced parts and steps of fabrication. This system has been successfully used for monolayer cultures, such as pheochromocytoma (PC-12) cells, reported by [30].

Human umbilical vein endothelial cells (HUVECs) represent a widely used source of primary endothelial cells for in vitro studies of the vasculature and angiogenesis. Although this model does not represent all endothelial cell types found in an organism, they are the most simple and available human EC type, accurate for the preparation of large quantities of cells [34,35]. Induction of HUVECs seeded in the 3D culture well of the device to form a vascular network was successful by seeding at an appropriate cell density and using the conditioned media of hLFs. First, we cocultured HUVECs and hLFs for vascular network formation. However, in most cases, hLFs dominate the culture volume and no visible lumen from HUVECs was observed. Although the comparability of angiogenesis induction by conditioned media and co-culture remains to be investigated, our experimental results show that hLF-conditioned medium works for angiogenesis induction at least as long as our PDMS-well/tubular reservoir system was used. Additionally, a cell density as high as 2 × 10^5^ cells/well was optimal for on-chip angiogenesis and conservation of the vasculature for a long time (10 days) under normoxic conditions.

The effect of hypoxia on the network formation from HUVECs depended on the duration of the preceding normoxic culture, or preincubation. Depending on the concentration and time of exposure, hypoxia can prevent the proliferation of cells and vessels or can act as a signal to trigger endothelial cells to begin the formation of new vasculature. In this regard, as soon as hypoxia is sensed by the endothelial cells, they activate their master oxygen sensors, the hypoxia inducible factors (HIFs). HIF-α activates and translocates into the endothelial cell nucleus and attaches to HIF-β. The complex then enters the angiogenic signaling pathway, modulating the formation of capillaries and vessels [5]. We did not have any knowledge about the normoxia/hypoxia relationship to maximize the lumen formation in our microdevice. The network formation and stability of the morphology of cells and lumens was nearly proportional to the preincubation time. Additionally, the combination of a long pre-incubation time and short hypoxia increased the width and length of the lumens and reduced the number of isolated elements in comparison with the normoxic culture for the same total duration. However, longer hypoxia affected the network stability and led to the disruption of lumens, while a long pre-incubation improved cell viability. Hypoxia is an external factor that plays a dual role in vascularization with both proangiogenic and vessel growth inhibition. Besides the time of hypoxic exposure, the transition between normoxic/hypoxic status could be the factor that determines the effect of hypoxia on endothelial cells. This result will give a new perspective that relates vasculogenesis and intermittent or episodic hypoxia. The use of normoxic/hypoxic transition to control the vascular network formation is a novel approach, but it still needs to be investigated. For that reason, we propose alternating periods (cycles) of normoxia/hypoxia in the networks cultured in our device and to evaluate which condition promotes the development of the vessel for long-term maintenance.

However, it is not clear how the normoxic/hypoxic system could improve the features of the circulatory system. Furthermore, more studies on biochemical mechanisms of the preincubation effect on vessel growth are required. The tracking of biochemical mechanisms in real-time imaging of 3D culture could be one potential application offered by this device.

In addition, our device is promising as a platform for the development of complex human organoids that contain microvasculature for better oxygen, nutrient, and metabolite exchange. Vascularization is considered one of the greatest challenges in the development of organoids [36,37]. Existing strategies to recreate vasculature including additive manufacturing based on photosensitive polymers and the use of sacrificial structures have limitations mainly in the removal of cytotoxicity [38,39]. Additionally, their considerable infrastructure cost precludes their wider adoption [3]. Our on-chip 3D hypoxia device will provide a platform for vasculature formation in organoids without cytotoxicity and in reasonably high throughput. Moreover, hypoxia is related to the angiogenic capability of cancer cells, and may mediate the progression of cancer. We could incorporate cells extracted from a biopsy into our device, and evaluate the angiogenic capacity to estimate their aggressiveness. Hypoxia is relevant in the treatment of some diseases such as cancer. For example, the effectiveness of radiotherapy relies on the oxygen radicals (ROS) produced by irradiating molecular oxygen [40]. Therefore, our device will also work as a platform for the therapeutic application of organoids.

## 5. Conclusions

We developed a portable standalone device to culture HUVECs under a 3D environment (fibrin–collagen gel) and expose them to transitions of normoxia/hypoxia. We confirmed that seeding at (1.59 ± 0.07) × 10^4^ cells/mm^2^ and the use of hLF-conditioned medium provide repeatable vascular network formation. We then evaluated the preincubation in normoxia as a parameter that influences the effect of hypoxia in the development and stability of vascular networks. The increased duration of preincubation (72 h) increases angiogenic parameters, and this condition favors the vascular promoting effect of hypoxia for 24 h.

The adaptation of endothelial cells to the transition of normoxia–hypoxia is an approach that allows for the formation and better development of a vascular network. For that reason, this device was important to maintain vascular networks in an easy configuration and introduces a different perspective of the study of cell cultures under hypoxic environments. Our result could potentially lead to the development of more advanced microdevices for cell culture (e.g., culture of organoids) that nowadays present technical difficulties for long-term maintenance. Particularly, hypoxia conditions will benefit the development of hypoxia-induced angiogenesis in organoid cultures or other kinds of biomimetic models.

## Figures and Tables

**Table 1:** The pH, temperature, and calculated pCO_2_ levels inside the inner reservoir of cell culture devices incubated on-chip and jacketed with bicarbonate buffer with or without sodium ascorbate.

Condition	pH	Temperature (°C)	pCO_2_ (%)
Bicarbonate (Normoxia)	7.13 ± 0.18	36.8 ± 0.06	5.19 ± 1.70
Bicarbonate + Ascorbate (Hypoxia)	7.11 ± 0.07	36.4 ± 0.05	5.22 ± 0.72

## Supplementary Materials

The following are available online at https://www.mdpi.com/2072-666X/11/5/475/s1, Figure S1: Schematic of the fabrication process for the microdevice; the fabrication steps carried out in the manufacture of the 3D cell culture device.

## References

[1] Bae H., Puranik A.S., Gauvin R., Edalat F., Carrillo-Conde B., Peppas N.A., Khademhosseini A. (2012). Building vascular networks. Sci. Transl. Med..

[2] Cochrane A., Albers H.J., Passier R., Mummery C.L., Van Den Berg A., Orlova V.V., Van Der Meer A.D. (2019). Advanced in vitro models of vascular biology: Human induced pluripotent stem cells and organ-on-chip technology. Adv. Drug Deliv. Rev..

[3] Grebenyuk S., Ranga A. (2019). Engineering Organoid Vascularization. Front. Bioeng. Biotechnol..

[4] Toussaint O., Weemaels G., Debacq-Chainiaux F., Scharffetter-Kochanek K., Wlaschek M. (2011). Artefactual effects of oxygen on cell culture models of cellular senescence and stem cell biology. J. Cell. Physiol..

[5] Krock B.L., Skuli N., Simon M.C. (2011). Hypoxia-induced angiogenesis: Good and evil. Genes Cancer.

[6] Cao H., Yu D., Yan X., Wang B., Yu Z., Song Y., Sheng L. (2019). Hypoxia destroys the microstructure of microtubules and causes dysfunction of endothelial cells via the PI3K/Stathmin1 pathway. Cell Biosci..

[7] Abe H., Semba H., Takeda N. (2017). The roles of hypoxia signaling in the pathogenesis of cardiovascular diseases. J. Atheroscler. Thromb..

[8] Eltzchig H., Carmeliet P. (2011). Hypoxia and inflammation. N. Engl. J. Med..

[9] Rankin E.B., Giaccia A.J. (2008). The role of hypoxia-inducible factors in tumorigenesis. Cell Death Differ..

[10] Baldea I., Teacoe I., Olteanu D.E., Vaida-Voievod C., Clichici A., Sirbu A., Filip G.A., Clichici S. (2018). Effects of different hypoxia degrees on endothelial cell cultures-Time course study. Mech. Ageing Dev..

[11] Wu J., Lei Z., Yu J. (2015). Hypoxia induces autophagy in human vascular endothelial cells in a hypoxia-inducible factor 1dependent manner. Mol. Med. Rep..

[12] Michiels C. (2004). Physiological and pathological responses to hypoxia. Am. J. Pathol..

[13] Tang N., Wang L., Esko J., Giordano F.J., Huang Y., Gerber H.P., Ferrara N., Johnson R.S. (2004). Loss of HIF-1α in endothelial cells disrupts a hypoxia-driven VEGF autocrine loop necessary for tumorigenesis. Cancer Cell.

[14] LaGory E.L., Giaccia A.J. (2016). The ever-expanding role of HIF in tumour and stromal biology. Nat. Cell Biol..

[15] Fathollahipour S., Patil P.S., Leipzig N.D. (2018). Oxygen Regulation in Development: Lessons from Embryogenesis towards Tissue Engineering. Cells Tissues Organs.

[16] Hutton D.L., Grayson W.L. (2016). Hypoxia inhibits de novo vascular assembly of adipose-derived stromal/stem cell populations, but promotes growth of preformed vessels. Tissue Eng. Part A.

[17] Nyberg E., Grayson W.L. (2018). Assessing the Minimum Time-Period of Normoxic Preincubation for Stable Adipose Stromal Cell-Derived Vascular Networks. Cell Mol. Bioeng..

[18] Byrne M.B., Leslie M.T., Gaskins H.R., Kenis P.J.A. (2014). Methods to study the tumor microenvironment under controlled oxygen conditions. Trends Biotechnol..

[19] Funamoto K., Zervantonakis I.K., Liu Y., Ochs C.J., Kim C., Kamm R.D. (2012). A novel microfluidic platform for high-resolution imaging of a three-dimensional cell culture under a controlled hypoxic environment. Lab. Chip.

[20] Bakmiwewa S.M., Heng B., Guillemin G.J., Ball H.J., Hunt N.H. (2015). An effective, low-cost method for achieving and maintaining hypoxia during cell culture studies. Biotechniques.

[21] Skolimowski M., Nielsen M.W., Emnéus J., Molin S., Taboryski R., Sternberg C., Dufva M., Geschke O. (2010). Microfluidic dissolved oxygen gradient generator biochip as a useful tool in bacterial biofilm studies. Lab. Chip.

[22] Chen Y.A., King A.D., Shih H.C., Peng C.C., Wu C.Y., Liao W.H., Tung Y.C. (2011). Generation of oxygen gradients in microfluidic devices for cell culture using spatially confined chemical reactions. Lab. Chip.

[23] Park J., Bansal T., Pinelis M., Maharbiz M.M. (2006). A microsystem for sensing and patterning oxidative microgradients during cell culture. Lab. Chip.

[24] Mosadegh B., Lockett M.R., Minn K.T., Simon K.A., Gilbert K., Hillier S., Newsome D., Li H., Hall A.B., Boucher D.M. (2015). A paper-based invasion assay: Assessing chemotaxis of cancer cells in gradients of oxygen. Biomaterials.

[25] Wu H.M., Lee T.A., Ko P.L., Chiang H.J., Peng C.C., Tung Y.C. (2018). Review of microfluidic cell culture devices for the control of gaseous microenvironments in vitro. J. Micromech. Microeng..

[26] Munoz-Sanchez J., Chanez-Cardenas M.E. (2019). The use of cobalt chloride as a chemical hypoxia model. J. Appl. Toxicol..

[27] Niu N., Li Z., Zhu M., Sun H., Yang J., Xu S., Zhao W., Song R. (2019). Effects of nuclear respiratory factor1 on apoptosis and mitochondrial dysfunction induced by cobalt chloride in H9C2 cells. Mol. Med. Rep..

[28] Reist M., Marshall K.A., Jenner P., Halliwell B. (1998). Toxic effects of sulphite in combination with peroxynitrite on neuronal cells. J. Neurochem..

[29] Vengellur A., Phillips J.M., Hogenesch J.B., LaPres J.J. (2005). Gene expression profiling of hypoxia signaling in human hepatocellular carcinoma cells. Physiol. Genom..

[30] Takano A., Tanaka M., Futai N. (2014). On-chip multi-gas incubation for microfluidic cell cultures under hypoxia. Biomicrofluidics.

[31] Carpentier G. Angiogenesis Analyzer for ImageJ. http://image.bio.methods.free.fr/ImageJ/?Angiogenesis-Analyzer-for-ImageJ.

[32] Kim S., Lee H., Chung M., Jeon N.L. (2013). Engineering of functional, perfusable 3D microvascular networks on a chip. Lab. Chip.

[33] Kim S., Chung M., Ahn J., Lee S., Jeon N.L. (2016). Interstitial flow regulates the angiogenic response and phenotype of endothelial cells in a 3D culture model. Lab. Chip.

[34] Baudin B., Bruneel A., Bosselut N., Vaubourdolle M. (2007). A protocol for isolation and culture of human umbilical vein endothelial cells. Nat. Protoc..

[35] Kocherova I., Bryja A., Mozdziak P., Angelova Volponi A., Dyszkiewicz-Konwińska M., Piotrowska-Kempisty H., Zabel M. (2019). Human Umbilical Vein Endothelial Cells (HUVECs) Co-Culture with Osteogenic Cells: From Molecular Communication to Engineering Prevascularised Bone Grafts. J. Clin. Med..

[36] Costa-Almeida R., Granja P.L., Soares R., Guerreiro S.G. (2014). Cellular strategies to promote vascularisation in tissue engineering applications. Eur. Cell Mater..

[37] Grassi L., Alfonsi R., Francescangeli F., Signore M., De Angelis M.L., Addario A., Costantini M., Flex E., Ciolfi A., Pizzi S. (2019). Organoids as a new model for improving regenerative medicine and cancer personalized therapy in renal diseases. Cell Death Dis..

[38] Wu W., DeConinck A., Lewis J.A. (2011). Omnidirectional printing of 3D microvascular networks. Adv. Mater..

[39] Li S., Liu Y.Y., Liu L.J., Hu Q.X. (2016). A Versatile Method for Fabricating Tissue Engineering Scaffolds with a Three-Dimensional Channel for Prevasculature Networks. ACS Appl. Mater. Interfaces.

[40] Metsala O., Kreutzer J., Hogel H., Miikkulainen P., Kallio P., Jaakkola P.M. (2018). Transportable system enabling multiple irradiation studies under simultaneous hypoxia in vitro. Radiat. Oncol..

