# Supplementary material for: Integrated On-Chip 3D Vascular Network Culture under Hypoxia
Source: Micromachines (Basel). 2020 Apr 30;11(5):475. doi: 10.3390/mi11050475 (PMC7281659; doi:10.3390/mi11050475)
Supplement: Supplementary file 1 [file micromachines-11-00475-s001.zip › micromachines-765381-supplementary-final/micromachines-765381-Supplementary Materials.docx]

[Supplementary](https://susy.mdpi.com/user/manuscripts/displayFile/46bc5eb7f3512304df83fcc097923ac8/supplementary) Materials:

Integrated On-Chip 3D Vascular Network Culture under Hypoxia

Miguel Ángel Olmedo-Suárez ^1^, Tomohiro Sekiguchi ^2^, Atsushi Takano ^3^, Maria del Pilar Cañizares-Macías ^1^, and Nobuyuki Futai ^2,^*

^1^ Departamento de Química Analítica, Facultad de Química, Universidad Nacional Autónoma de México, Av. Universidad 3000, Ciudad de México 04510, México; miolsu22@hotmail.com (M.Á.O.-S.); pilarm@unam.mx (M.d.P.C.-M.)

^2^ Department of Mechanical Engineering, College of Engineering, Shibaura Institute of Technology, 3-7-5 Toyosu, Koto-ku, Tokyo 135-8548, Japan; md18048@shibaura-it.ac.jp

^3^ Digital Manufacturing and Design Centre, Singapore University of Technology and Design, 8 Somapah Road, Singapore 487372, Singapore; cookdo812@hotmail.com

***** Correspondence: futai@shibaura-it.ac.jp; Tel.: +81-3-5859-8016

**
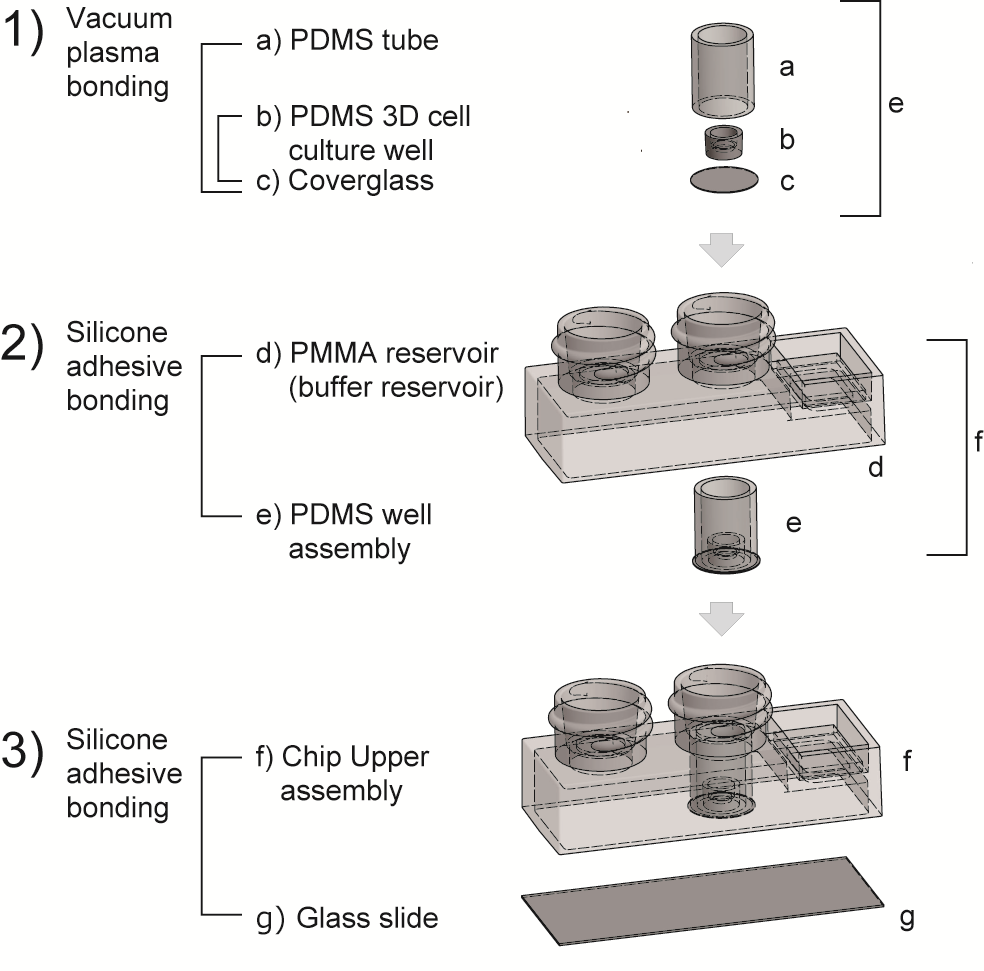
**

**Figure S1.** Schematic of the fabrication process for the microdevice.

| 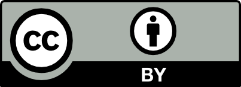 | © 2020 by the authors. Submitted for possible open access publication under the terms and conditions of the Creative Commons Attribution (CC BY) license (http://creativecommons.org/licenses/by/4.0/). |
| --- | --- |
